# 2,6-Bis(4-meth­oxy­phen­yl)-1,3-dimethyl­piperidin-4-one *O*-benzyl­oxime

**DOI:** 10.1107/S1600536812002140

**Published:** 2012-01-25

**Authors:** Dong Ho Park, V. Ramkumar, P. Parthiban

**Affiliations:** a Department of Biomedicinal Chemistry, Inje University, Gimhae, Gyeongnam 621 749, Republic of Korea; b Department of Chemistry, IIT Madras, Chennai 600 036, TamilNadu, India

## Abstract

The central ring of the title compound, C_28_H_32_N_2_O_3_, exists in a chair conformation with an equatorial disposition of all the alkyl and aryl groups on the heterocycle. The *para*-anisyl groups on both sides of the secondary amino group are oriented at an angle of 54.75 (4)° with respect to each other. The oxime derivative exists as an *E* isomer with the methyl substitution on one of the active methyl­ene centers of the mol­ecule. The crystal packing features weak C—H⋯O inter­actions.

## Related literature

For the synthesis and biological activity of piperidin-4-ones, see: Parthiban *et al.* (2005 ▶, 2008 ▶, 2009*a* ▶, 2011 ▶). For related structures, see: Parthiban *et al.* (2009*a* ▶,*b* ▶); For ring puckering parameters, see: Cremer & Pople (1975 ▶); Nardelli (1983 ▶).
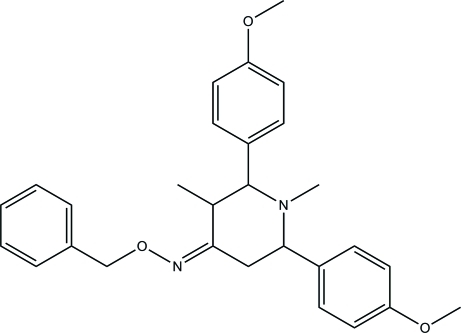


## Experimental

### 

#### Crystal data

C_28_H_32_N_2_O_3_
*M* *_r_* = 444.56
Monoclinic, 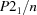

*a* = 16.674 (5) Å
*b* = 19.819 (8) Å
*c* = 7.549 (1) Å
β = 90.080 (5)°
*V* = 2494.7 (13) Å^3^
*Z* = 4
Mo *K*α radiation
μ = 0.08 mm^−1^
*T* = 298 K
0.40 × 0.20 × 0.20 mm

#### Data collection

Bruker APEXII CCD area-detector diffractometer
Absorption correction: multi-scan (*SADABS*; Bruker, 2004 ▶) *T*_min_ = 0.970, *T*_max_ = 0.985
58357 measured reflections
6743 independent reflections
4156 reflections with *I* > 2σ(*I*)
*R*_int_ = 0.037

#### Refinement

*R*[*F* ^2^ > 2σ(*F* ^2^)] = 0.048
*wR*(*F* ^2^) = 0.160
*S* = 1.02
6743 reflections
302 parameters
H-atom parameters constrained
Δρ_max_ = 0.25 e Å^−3^
Δρ_min_ = −0.19 e Å^−3^

### 

Data collection: *APEX2* (Bruker, 2004 ▶); cell refinement: *APEX2* and *SAINT* (Bruker, 2004 ▶); data reduction: *SAINT* and *XPREP* (Bruker, 2004 ▶); program(s) used to solve structure: *SIR92* (Altomare *et al.*, 1993 ▶); program(s) used to refine structure: *SHELXL97* (Sheldrick, 2008 ▶); molecular graphics: *ORTEP-3* (Farrugia, 1997 ▶); software used to prepare material for publication: *SHELXL97*.

## Supplementary Material

Crystal structure: contains datablock(s) global, I. DOI: 10.1107/S1600536812002140/bq2334sup1.cif

Structure factors: contains datablock(s) I. DOI: 10.1107/S1600536812002140/bq2334Isup2.hkl

Supplementary material file. DOI: 10.1107/S1600536812002140/bq2334Isup3.cml

Additional supplementary materials:  crystallographic information; 3D view; checkCIF report

## Figures and Tables

**Table 1:** Hydrogen-bond geometry (Å, °)

*D*—H⋯*A*	*D*—H	H⋯*A*	*D*⋯*A*	*D*—H⋯*A*
C8—H8⋯O2^i^	0.93	2.51	3.340 (2)	149 (6)

Symmetry code: (i) 
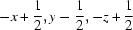
.

**Fig. 1.**
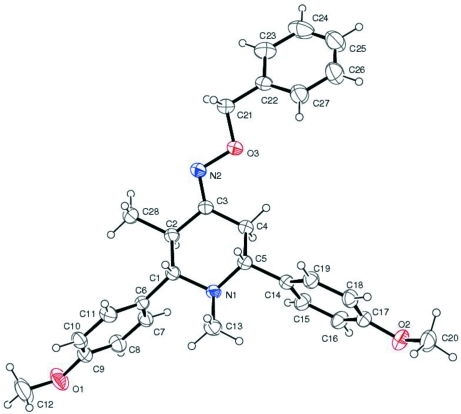
Molecular structure of the title compound, showing the atom-labeling scheme. Displacement ellipsoids are drawn at the 30% probability level

**Fig. 2.**
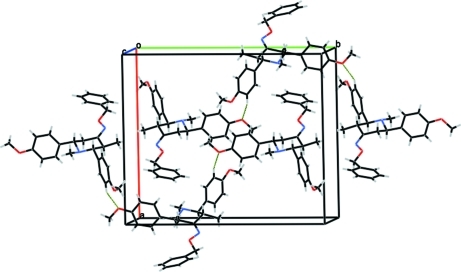
Molecular packing of the title compound. Hydrogen bonds are shown as dashed lines.

## supplementary crystallographic information

### Comment

Piperidone molecule is an important class of pharmacophore due to its broad-spectrum of biological actions ranging from antibacterial to anticancer (Parthiban *et al.*, 2005, 2009*a*, 2011). Because of its broad-spectrum of biological actions, isolation from the natural products as well as synthesis of new molecules, and their stereochemical analysis are continuously interested and important in the field of medicinal chemistry.

Hence, we synthesized the title compound by a successive double Mannich condensation to obtain the piperidin-4-one, which was further condensed with *O*-benzylhydroxylamine hydrochloride to make the oxime ether derivative of the piperidone. Thus the obtained crystal of the unsymmetrical molecule was undertaken for this study to explore its stereochemistry in the solid-state, since the *E/Z* isomerization plays a major role during oximation.

The XRD data of the title compound witnessed that the piperidone ring N1—C1—C2—C3—C4—C5 adopts a chair conformation with the deviation of ring atoms N1 and C3 from the best plane C1—C2—C4—C5 by -0.593 and 0.683 Å, respectively. According to Nardelli (Nardelli, 1983), the smallest displacement asymmetry parameters q_2_ and q_3_ are 0.084 (16) and 0.545 (16) Å, respectively. According to Cremer and Pople (Cremer & Pople, 1975), the ring puckering parameters such as total puckering amplitude Q_T_ and phase angle θ are 0.552 (16) Å and 171.18 (17)°. Thus, all parameters strongly support the chair conformation of the piperidone ring.

The torsion angles of C3—C2—C1—C6 and C3—C4—C5—C14 of the anisyl rings are 171.52 (3)° and 177.97 (3)°, and they are orientated at an angle of 57.41 (2)° with respect to each other. The crystal packing is stabilized by weak C—H···O interactions (Table 1).

### Experimental

The 2,6-*bis*(4-methoxyphenyl)-1,3-dimethylpiperidin-4-one *O*-benzyloxime was synthesized by one-pot using *para* anisaldehyde (0.1 mol, 13.61 g, 12.12 ml), 2-butanone (0.05 mol, 3.61 g, 4.48 ml) and ammonium acetate (0.05 mol, 3.85 g) in a 50 ml of absolute ethanol. The mixture was gently warmed on a hot plate at 303–308 K (30–35° C) with moderate stirring till the complete consumption of the starting materials, which was monitored by TLC. At the end, the crude piperidin-4-one was separated by filtration and gently washed with 1:5 cold ethanol-ether mixture. Then the pure product was *N*-methylated by methyl iodide using anhydrous potassium carbonate in dry acetone. Thus the obtained *N*-methylpiperidin-4-one (0.005 mol, 2.218 g) was condensed with *O*-benzylhydroxylamine hydrochloride (0.005 mol, 0.798 g) using sodium acetate trihydrate (0.015 mol, 2.04 g) as a base in methanol (Parthiban *et al.*, 2008, 2009). X-ray diffraction quality crystals of the title compound were obtained by slow evaporation from ethanol.

### Refinement

All hydrogen atoms were fixed geometrically and allowed to ride on the parent carbon atoms with aromatic C—H = 0.93 Å, methylene C—H = 0.97 Å, methine C—H = 0.98 Å and methyl C—H = 0.96 Å. The displacement parameters were set for phenyl, methylene and aliphatic H atoms at *U*_iso_(H) = 1.2*U*_eq_(C) and for methyl H atoms at*U*_iso_(H) = 1.5*U*_eq_(C)

### Crystal data

**Table:**

C_28_H_32_N_2_O_3_	*F*(000) = 952
*M**_r_* = 444.56	*D*_x_ = 1.184 Mg m^−^^3^
Monoclinic, *P*2_1_/*n*	Mo *K*α radiation, λ = 0.71069 Å
Hall symbol: -P 2yn	Cell parameters from 6328 reflections
*a* = 16.674 (5) Å	θ = 2.5–26.2°
*b* = 19.819 (8) Å	µ = 0.08 mm^−^^1^
*c* = 7.549 (1) Å	*T* = 298 K
β = 90.080 (5)°	Needle, colourless
*V* = 2494.7 (13) Å^3^	0.40 × 0.20 × 0.20 mm
*Z* = 4	

### Data collection

**Table:**

Bruker APEXII CCD area-detector diffractometer	6743 independent reflections
Radiation source: fine-focus sealed tube	4156 reflections with *I* > 2σ(*I*)
graphite	*R*_int_ = 0.037
ω and φ scan	θ_max_ = 29.2°, θ_min_ = 1.6°
Absorption correction: multi-scan (*SADABS*; Bruker, 2004)	*h* = −22→22
*T*_min_ = 0.970, *T*_max_ = 0.985	*k* = −27→27
58357 measured reflections	*l* = −10→10

### Refinement

**Table:**

Refinement on *F*^2^	Primary atom site location: structure-invariant direct methods
Least-squares matrix: full	Secondary atom site location: difference Fourier map
*R*[*F*^2^ > 2σ(*F*^2^)] = 0.048	Hydrogen site location: inferred from neighbouring sites
*wR*(*F*^2^) = 0.160	H-atom parameters constrained
*S* = 1.02	*w* = 1/[σ^2^(*F*_o_^2^) + (0.0731*P*)^2^ + 0.4684*P*] where *P* = (*F*_o_^2^ + 2*F*_c_^2^)/3
6743 reflections	(Δ/σ)_max_ < 0.001
302 parameters	Δρ_max_ = 0.25 e Å^−^^3^
0 restraints	Δρ_min_ = −0.19 e Å^−^^3^

### Special details

**Table:**

Geometry. All e.s.d.'s (except the e.s.d. in the dihedral angle between two l.s. planes) are estimated using the full covariance matrix. The cell e.s.d.'s are taken into account individually in the estimation of e.s.d.'s in distances, angles and torsion angles; correlations between e.s.d.'s in cell parameters are only used when they are defined by crystal symmetry. An approximate (isotropic) treatment of cell e.s.d.'s is used for estimating e.s.d.'s involving l.s. planes.
Refinement. Refinement of *F*^2^ against ALL reflections. The weighted *R*-factor *wR* and goodness of fit *S* are based on *F*^2^, conventional *R*-factors *R* are based on *F*, with *F* set to zero for negative *F*^2^. The threshold expression of *F*^2^ > σ(*F*^2^) is used only for calculating *R*-factors(gt) *etc*. and is not relevant to the choice of reflections for refinement. *R*-factors based on *F*^2^ are statistically about twice as large as those based on *F*, and *R*- factors based on ALL data will be even larger.

### Fractional atomic coordinates and isotropic or equivalent isotropic displacement parameters (Å^2^)

**Table:**

	*x*	*y*	*z*	*U*_iso_*/*U*_eq_	
C1	0.44194 (8)	0.16166 (7)	0.11676 (18)	0.0417 (3)	
H1	0.4945	0.1589	0.0592	0.050*	
C2	0.44660 (10)	0.12323 (7)	0.29407 (19)	0.0470 (3)	
H2	0.3920	0.1214	0.3419	0.056*	
C3	0.49689 (9)	0.16058 (7)	0.42603 (19)	0.0466 (3)	
C4	0.47747 (11)	0.23373 (8)	0.4419 (2)	0.0571 (4)	
H4A	0.5160	0.2554	0.5195	0.068*	
H4B	0.4246	0.2391	0.4933	0.068*	
C5	0.47965 (9)	0.26701 (7)	0.2596 (2)	0.0457 (3)	
H5	0.5335	0.2611	0.2101	0.055*	
C6	0.38096 (8)	0.12780 (7)	−0.00204 (18)	0.0397 (3)	
C7	0.30053 (9)	0.12442 (8)	0.0456 (2)	0.0487 (4)	
H7	0.2837	0.1451	0.1496	0.058*	
C8	0.24547 (9)	0.09114 (9)	−0.0577 (2)	0.0566 (4)	
H8	0.1921	0.0891	−0.0228	0.068*	
C9	0.26938 (10)	0.06092 (8)	−0.2127 (2)	0.0549 (4)	
C10	0.34798 (10)	0.06385 (9)	−0.2638 (2)	0.0575 (4)	
H10	0.3643	0.0435	−0.3687	0.069*	
C11	0.40321 (9)	0.09731 (9)	−0.1580 (2)	0.0513 (4)	
H11	0.4566	0.0992	−0.1934	0.062*	
C12	0.23105 (18)	−0.00445 (16)	−0.4616 (4)	0.1228 (11)	
H12A	0.2481	0.0276	−0.5491	0.184*	
H12B	0.1856	−0.0291	−0.5054	0.184*	
H12C	0.2740	−0.0353	−0.4367	0.184*	
C13	0.42251 (12)	0.26663 (9)	−0.0327 (2)	0.0646 (5)	
H13A	0.4064	0.3129	−0.0199	0.097*	
H13B	0.3860	0.2438	−0.1109	0.097*	
H13C	0.4757	0.2648	−0.0810	0.097*	
C14	0.46296 (9)	0.34154 (7)	0.27568 (19)	0.0444 (3)	
C15	0.38889 (9)	0.36556 (8)	0.3302 (2)	0.0550 (4)	
H15	0.3486	0.3351	0.3594	0.066*	
C16	0.37405 (10)	0.43351 (8)	0.3418 (2)	0.0574 (4)	
H16	0.3238	0.4486	0.3774	0.069*	
C17	0.43340 (10)	0.47954 (7)	0.3007 (2)	0.0488 (4)	
C18	0.50806 (10)	0.45703 (8)	0.2520 (2)	0.0517 (4)	
H18	0.5488	0.4876	0.2273	0.062*	
C19	0.52203 (9)	0.38844 (8)	0.2399 (2)	0.0492 (4)	
H19	0.5727	0.3734	0.2067	0.059*	
C20	0.46999 (14)	0.59501 (9)	0.2670 (3)	0.0741 (6)	
H20A	0.5144	0.5914	0.3474	0.111*	
H20B	0.4468	0.6392	0.2762	0.111*	
H20C	0.4883	0.5877	0.1480	0.111*	
C21	0.64597 (11)	0.13411 (9)	0.7318 (3)	0.0648 (5)	
H21A	0.6908	0.1253	0.6535	0.078*	
H21B	0.6247	0.0912	0.7719	0.078*	
C22	0.67301 (9)	0.17495 (8)	0.8864 (2)	0.0495 (4)	
C23	0.68344 (12)	0.14580 (12)	1.0497 (3)	0.0741 (5)	
H23	0.6730	0.1001	1.0665	0.089*	
C24	0.71023 (16)	0.18628 (18)	1.1919 (3)	0.1016 (9)	
H24	0.7175	0.1673	1.3034	0.122*	
C25	0.72539 (15)	0.25289 (17)	1.1660 (4)	0.1004 (8)	
H25	0.7437	0.2792	1.2597	0.120*	
C26	0.71426 (14)	0.28085 (13)	1.0079 (4)	0.0919 (7)	
H26	0.7239	0.3267	0.9921	0.110*	
C27	0.68888 (12)	0.24268 (10)	0.8690 (3)	0.0692 (5)	
H27	0.6821	0.2630	0.7589	0.083*	
C28	0.47333 (13)	0.05063 (8)	0.2641 (2)	0.0679 (5)	
H28A	0.5263	0.0503	0.2142	0.102*	
H28B	0.4368	0.0288	0.1841	0.102*	
H28C	0.4738	0.0270	0.3751	0.102*	
N1	0.42166 (7)	0.23357 (6)	0.14133 (16)	0.0428 (3)	
N2	0.54935 (8)	0.12812 (7)	0.51302 (17)	0.0507 (3)	
O1	0.20983 (8)	0.02954 (9)	−0.3066 (2)	0.0900 (5)	
O2	0.41151 (8)	0.54589 (6)	0.31036 (17)	0.0671 (3)	
O3	0.58612 (7)	0.17071 (6)	0.64134 (15)	0.0599 (3)	

### Atomic displacement parameters (Å^2^)

**Table:**

	*U*^11^	*U*^22^	*U*^33^	*U*^12^	*U*^13^	*U*^23^
C1	0.0424 (7)	0.0405 (8)	0.0424 (7)	−0.0023 (6)	−0.0045 (6)	0.0015 (6)
C2	0.0558 (9)	0.0408 (8)	0.0443 (8)	−0.0030 (6)	−0.0102 (7)	0.0040 (6)
C3	0.0579 (9)	0.0426 (8)	0.0394 (7)	0.0024 (7)	−0.0089 (7)	0.0033 (6)
C4	0.0757 (11)	0.0461 (9)	0.0492 (9)	0.0079 (8)	−0.0209 (8)	−0.0037 (7)
C5	0.0457 (8)	0.0398 (8)	0.0518 (9)	0.0000 (6)	−0.0103 (6)	−0.0004 (6)
C6	0.0435 (7)	0.0367 (7)	0.0390 (7)	−0.0007 (6)	−0.0033 (6)	0.0022 (6)
C7	0.0471 (8)	0.0544 (9)	0.0447 (8)	0.0018 (7)	0.0003 (6)	−0.0085 (7)
C8	0.0413 (8)	0.0672 (11)	0.0614 (10)	−0.0037 (7)	−0.0005 (7)	−0.0080 (8)
C9	0.0520 (9)	0.0528 (9)	0.0597 (10)	0.0005 (7)	−0.0154 (7)	−0.0117 (8)
C10	0.0621 (10)	0.0671 (11)	0.0432 (8)	0.0105 (8)	−0.0061 (7)	−0.0150 (8)
C11	0.0451 (8)	0.0657 (10)	0.0432 (8)	0.0030 (7)	0.0032 (6)	−0.0023 (7)
C12	0.122 (2)	0.135 (2)	0.111 (2)	0.0023 (18)	−0.0431 (17)	−0.0718 (19)
C13	0.0899 (13)	0.0499 (9)	0.0539 (10)	−0.0123 (9)	−0.0201 (9)	0.0115 (8)
C14	0.0456 (8)	0.0405 (7)	0.0470 (8)	−0.0018 (6)	−0.0100 (6)	0.0002 (6)
C15	0.0465 (8)	0.0436 (8)	0.0748 (11)	−0.0044 (7)	−0.0034 (8)	0.0069 (8)
C16	0.0486 (9)	0.0492 (9)	0.0745 (11)	0.0054 (7)	0.0019 (8)	0.0050 (8)
C17	0.0603 (9)	0.0385 (8)	0.0477 (8)	0.0005 (7)	−0.0052 (7)	0.0018 (6)
C18	0.0556 (9)	0.0449 (8)	0.0546 (9)	−0.0098 (7)	0.0001 (7)	0.0005 (7)
C19	0.0459 (8)	0.0456 (8)	0.0560 (9)	−0.0020 (6)	−0.0014 (7)	−0.0014 (7)
C20	0.1086 (16)	0.0404 (9)	0.0735 (12)	−0.0087 (10)	−0.0008 (11)	0.0028 (8)
C21	0.0665 (11)	0.0592 (10)	0.0687 (11)	0.0128 (8)	−0.0259 (9)	−0.0051 (9)
C22	0.0391 (7)	0.0592 (10)	0.0503 (9)	0.0025 (6)	−0.0074 (6)	0.0014 (7)
C23	0.0703 (12)	0.0867 (14)	0.0652 (12)	0.0025 (10)	−0.0093 (9)	0.0164 (11)
C24	0.1032 (18)	0.153 (3)	0.0488 (12)	0.0167 (18)	−0.0201 (12)	0.0042 (14)
C25	0.0884 (17)	0.118 (2)	0.0948 (19)	0.0112 (15)	−0.0279 (14)	−0.0426 (17)
C26	0.0855 (15)	0.0815 (15)	0.109 (2)	−0.0083 (12)	−0.0196 (14)	−0.0250 (14)
C27	0.0736 (12)	0.0656 (12)	0.0685 (12)	−0.0081 (9)	−0.0072 (9)	−0.0015 (9)
C28	0.0986 (14)	0.0398 (9)	0.0652 (11)	0.0002 (9)	−0.0293 (10)	0.0032 (8)
N1	0.0487 (7)	0.0369 (6)	0.0429 (6)	−0.0036 (5)	−0.0111 (5)	0.0052 (5)
N2	0.0596 (8)	0.0475 (7)	0.0449 (7)	−0.0007 (6)	−0.0140 (6)	0.0013 (6)
O1	0.0690 (8)	0.1054 (12)	0.0954 (11)	−0.0067 (8)	−0.0256 (8)	−0.0440 (9)
O2	0.0817 (9)	0.0394 (6)	0.0804 (9)	0.0047 (6)	0.0045 (7)	0.0024 (6)
O3	0.0722 (8)	0.0498 (6)	0.0576 (7)	0.0071 (5)	−0.0286 (6)	−0.0039 (5)

### Geometric parameters (Å, °)

**Table:**

C1—N1	1.4765 (19)	C14—C19	1.381 (2)
C1—C6	1.5119 (19)	C14—C15	1.387 (2)
C1—C2	1.542 (2)	C15—C16	1.372 (2)
C1—H1	0.9800	C15—H15	0.9300
C2—C3	1.497 (2)	C16—C17	1.381 (2)
C2—C28	1.523 (2)	C16—H16	0.9300
C2—H2	0.9800	C17—O2	1.3667 (19)
C3—N2	1.2684 (19)	C17—C18	1.373 (2)
C3—C4	1.490 (2)	C18—C19	1.382 (2)
C4—C5	1.526 (2)	C18—H18	0.9300
C4—H4A	0.9700	C19—H19	0.9300
C4—H4B	0.9700	C20—O2	1.417 (2)
C5—N1	1.4729 (18)	C20—H20A	0.9600
C5—C14	1.508 (2)	C20—H20B	0.9600
C5—H5	0.9800	C20—H20C	0.9600
C6—C11	1.375 (2)	C21—O3	1.4095 (19)
C6—C7	1.391 (2)	C21—C22	1.490 (2)
C7—C8	1.372 (2)	C21—H21A	0.9700
C7—H7	0.9300	C21—H21B	0.9700
C8—C9	1.374 (2)	C22—C23	1.372 (2)
C8—H8	0.9300	C22—C27	1.374 (3)
C9—C10	1.368 (2)	C23—C24	1.412 (3)
C9—O1	1.369 (2)	C23—H23	0.9300
C10—C11	1.387 (2)	C24—C25	1.358 (4)
C10—H10	0.9300	C24—H24	0.9300
C11—H11	0.9300	C25—C26	1.329 (4)
C12—O1	1.396 (3)	C25—H25	0.9300
C12—H12A	0.9600	C26—C27	1.360 (3)
C12—H12B	0.9600	C26—H26	0.9300
C12—H12C	0.9600	C27—H27	0.9300
C13—N1	1.468 (2)	C28—H28A	0.9600
C13—H13A	0.9600	C28—H28B	0.9600
C13—H13B	0.9600	C28—H28C	0.9600
C13—H13C	0.9600	N2—O3	1.4231 (16)
			
N1—C1—C6	110.43 (11)	C15—C14—C5	121.64 (13)
N1—C1—C2	112.27 (12)	C16—C15—C14	121.10 (15)
C6—C1—C2	109.19 (11)	C16—C15—H15	119.4
N1—C1—H1	108.3	C14—C15—H15	119.4
C6—C1—H1	108.3	C15—C16—C17	120.31 (15)
C2—C1—H1	108.3	C15—C16—H16	119.8
C3—C2—C28	113.71 (13)	C17—C16—H16	119.8
C3—C2—C1	111.16 (12)	O2—C17—C18	124.69 (14)
C28—C2—C1	110.61 (13)	O2—C17—C16	115.60 (15)
C3—C2—H2	107.0	C18—C17—C16	119.70 (15)
C28—C2—H2	107.0	C17—C18—C19	119.34 (14)
C1—C2—H2	107.0	C17—C18—H18	120.3
N2—C3—C4	126.98 (14)	C19—C18—H18	120.3
N2—C3—C2	118.62 (13)	C14—C19—C18	121.92 (15)
C4—C3—C2	114.38 (13)	C14—C19—H19	119.0
C3—C4—C5	110.03 (13)	C18—C19—H19	119.0
C3—C4—H4A	109.7	O2—C20—H20A	109.5
C5—C4—H4A	109.7	O2—C20—H20B	109.5
C3—C4—H4B	109.7	H20A—C20—H20B	109.5
C5—C4—H4B	109.7	O2—C20—H20C	109.5
H4A—C4—H4B	108.2	H20A—C20—H20C	109.5
N1—C5—C14	111.62 (12)	H20B—C20—H20C	109.5
N1—C5—C4	109.62 (12)	O3—C21—C22	108.26 (14)
C14—C5—C4	110.26 (13)	O3—C21—H21A	110.0
N1—C5—H5	108.4	C22—C21—H21A	110.0
C14—C5—H5	108.4	O3—C21—H21B	110.0
C4—C5—H5	108.4	C22—C21—H21B	110.0
C11—C6—C7	117.50 (13)	H21A—C21—H21B	108.4
C11—C6—C1	121.40 (13)	C23—C22—C27	118.24 (17)
C7—C6—C1	121.07 (13)	C23—C22—C21	120.85 (17)
C8—C7—C6	121.40 (14)	C27—C22—C21	120.91 (16)
C8—C7—H7	119.3	C22—C23—C24	118.9 (2)
C6—C7—H7	119.3	C22—C23—H23	120.6
C7—C8—C9	119.90 (15)	C24—C23—H23	120.6
C7—C8—H8	120.1	C25—C24—C23	120.1 (2)
C9—C8—H8	120.1	C25—C24—H24	120.0
C10—C9—O1	124.59 (15)	C23—C24—H24	120.0
C10—C9—C8	120.06 (14)	C26—C25—C24	120.6 (2)
O1—C9—C8	115.35 (15)	C26—C25—H25	119.7
C9—C10—C11	119.57 (15)	C24—C25—H25	119.7
C9—C10—H10	120.2	C25—C26—C27	120.2 (2)
C11—C10—H10	120.2	C25—C26—H26	119.9
C6—C11—C10	121.57 (14)	C27—C26—H26	119.9
C6—C11—H11	119.2	C26—C27—C22	121.9 (2)
C10—C11—H11	119.2	C26—C27—H27	119.0
O1—C12—H12A	109.5	C22—C27—H27	119.0
O1—C12—H12B	109.5	C2—C28—H28A	109.5
H12A—C12—H12B	109.5	C2—C28—H28B	109.5
O1—C12—H12C	109.5	H28A—C28—H28B	109.5
H12A—C12—H12C	109.5	C2—C28—H28C	109.5
H12B—C12—H12C	109.5	H28A—C28—H28C	109.5
N1—C13—H13A	109.5	H28B—C28—H28C	109.5
N1—C13—H13B	109.5	C13—N1—C5	109.55 (12)
H13A—C13—H13B	109.5	C13—N1—C1	108.42 (12)
N1—C13—H13C	109.5	C5—N1—C1	111.11 (11)
H13A—C13—H13C	109.5	C3—N2—O3	110.35 (12)
H13B—C13—H13C	109.5	C9—O1—C12	117.96 (18)
C19—C14—C15	117.56 (14)	C17—O2—C20	117.71 (14)
C19—C14—C5	120.78 (14)	C21—O3—N2	109.19 (12)
			
N1—C1—C2—C3	−48.71 (17)	C15—C16—C17—O2	177.69 (16)
C6—C1—C2—C3	−171.52 (12)	C15—C16—C17—C18	−1.4 (3)
N1—C1—C2—C28	−176.03 (13)	O2—C17—C18—C19	−177.25 (15)
C6—C1—C2—C28	61.16 (17)	C16—C17—C18—C19	1.8 (2)
C28—C2—C3—N2	−7.6 (2)	C15—C14—C19—C18	−2.0 (2)
C1—C2—C3—N2	−133.25 (15)	C5—C14—C19—C18	179.39 (14)
C28—C2—C3—C4	173.41 (15)	C17—C18—C19—C14	−0.1 (2)
C1—C2—C3—C4	47.80 (19)	O3—C21—C22—C23	135.37 (17)
N2—C3—C4—C5	128.05 (17)	O3—C21—C22—C27	−45.2 (2)
C2—C3—C4—C5	−53.11 (19)	C27—C22—C23—C24	−0.3 (3)
C3—C4—C5—N1	58.77 (18)	C21—C22—C23—C24	179.12 (19)
C3—C4—C5—C14	−177.97 (13)	C22—C23—C24—C25	−0.1 (4)
N1—C1—C6—C11	121.01 (15)	C23—C24—C25—C26	0.9 (4)
C2—C1—C6—C11	−115.08 (16)	C24—C25—C26—C27	−1.2 (4)
N1—C1—C6—C7	−61.16 (17)	C25—C26—C27—C22	0.8 (4)
C2—C1—C6—C7	62.75 (18)	C23—C22—C27—C26	0.0 (3)
C11—C6—C7—C8	0.9 (2)	C21—C22—C27—C26	−179.44 (19)
C1—C6—C7—C8	−177.04 (15)	C14—C5—N1—C13	56.26 (18)
C6—C7—C8—C9	−0.7 (3)	C4—C5—N1—C13	178.72 (13)
C7—C8—C9—C10	0.2 (3)	C14—C5—N1—C1	176.03 (12)
C7—C8—C9—O1	−179.49 (16)	C4—C5—N1—C1	−61.51 (16)
O1—C9—C10—C11	179.78 (17)	C6—C1—N1—C13	−60.66 (15)
C8—C9—C10—C11	0.2 (3)	C2—C1—N1—C13	177.22 (13)
C7—C6—C11—C10	−0.5 (2)	C6—C1—N1—C5	178.89 (12)
C1—C6—C11—C10	177.36 (15)	C2—C1—N1—C5	56.78 (16)
C9—C10—C11—C6	0.0 (3)	C4—C3—N2—O3	3.6 (2)
N1—C5—C14—C19	−125.45 (15)	C2—C3—N2—O3	−175.15 (13)
C4—C5—C14—C19	112.46 (16)	C10—C9—O1—C12	3.0 (3)
N1—C5—C14—C15	56.0 (2)	C8—C9—O1—C12	−177.4 (2)
C4—C5—C14—C15	−66.13 (19)	C18—C17—O2—C20	0.0 (2)
C19—C14—C15—C16	2.3 (2)	C16—C17—O2—C20	−179.11 (16)
C5—C14—C15—C16	−179.03 (15)	C22—C21—O3—N2	−169.64 (13)
C14—C15—C16—C17	−0.7 (3)	C3—N2—O3—C21	−178.00 (15)

### Hydrogen-bond geometry (Å, °)

**Table:**

*D*—H···*A*	*D*—H	H···*A*	*D*···*A*	*D*—H···*A*
C8—H8···O2^i^	0.93	2.51	3.340 (2)	149 (6)

Symmetry codes: (i) −*x*+1/2, *y*−1/2, −*z*+1/2.
